# Identifying Phase-Amplitude Coupling in Cyclic Alternating Pattern using Masking Signals

**DOI:** 10.1038/s41598-018-21013-9

**Published:** 2018-02-08

**Authors:** Chien-Hung Yeh, Wenbin Shi

**Affiliations:** 1 Department of Neurology, Chang Gung Memorial Hospital and University, Taoyuan City, Taiwan; 2 Department of Hydraulic Engineering, State Key Laboratory of Hydroscience and Engineering, Tsinghua University, Beijing, China

## Abstract

Judiciously classifying phase-A subtypes in cyclic alternating pattern (CAP) is critical for investigating sleep dynamics. Phase-amplitude coupling (PAC), one of the representative forms of neural rhythmic interaction, is defined as the amplitude of high-frequency activities modulated by the phase of low-frequency oscillations. To examine PACs under more or less synchronized conditions, we propose a nonlinear approach, named the masking phase-amplitude coupling (MPAC), to quantify physiological interactions between high (*α*/low*β*) and low (*δ*) frequency bands. The results reveal that the coupling intensity is generally the highest in subtype A1 and lowest in A3. MPACs among various physiological conditions/disorders (*p* < 0.0001) and sleep stages (*p* < 0.0001 except S4) are tested. MPACs are found significantly stronger in light sleep than deep sleep (*p* < 0.0001). Physiological conditions/disorders show similar order in MPACs. Phase-amplitude dependence between *δ* and *α*/low*β* oscillations are examined as well. *δ* phase tent to phase-locked to *α/*low*β* amplitude in subtype A1 more than the rest. These results suggest that an elevated *δ*-*α*/low*β* MPACs can reflect some synchronization in CAP. Therefore, MPAC can be a potential tool to investigate neural interactions between different time scales, and *δ*-*α*/low*β* MPAC can serve as a feasible biomarker for sleep microstructure.

## Introduction

Electroencephalograph (EEG) provides precious information about sleep dynamics in a non-invasive way. Sleep macrostructure, defined as the stepwise profile which classifies sleep stages according to the prevalent EEGs in consecutive 30-sec epochs, is widely-used as the conventional approach for sleep studies. Gradually, phasic EEG events observed during NREM sleep stages, which are shorter than the standardized scoring epoch, are taken into account. Identification of the features in cyclic alternating pattern (CAP) sequences can contribute to the development of sleep microstructural analysis. The definition of CAP sequence and phase-A subtypes are provided in the Supplementary Information. The importance of CAP demonstrates on its ability in translating the brain effort to regulate and preserve sleep macrostructure, and associate a condition of instability of the level of vigilance. In specific, subtype A1 usually contribute to the build-up and consolidation of deep sleep, whereas subtype A2 and A3 are involved in the onset of REM sleep^1^.

The most straightforward CAP-scoring method is visual recognition of the EEG waveform. Classical features for CAP scoring is described in detail by Parma’s group^2^. The visual assessment of sleep microstructure is time consuming and may deviate in a certain degree of inter-scorer agreement spanning from 69% to 77%^3^. Some studies introduced methods, mostly rely on the spectral assessments from the EEGs with the application of machine-learning algorithms, for the detection of CAP^4,5^. However, none of these methods are applied in clinics since they either require certain clinical intervention and/or large data volumes to provide the physiological information in need. Instead of concentrating on the characteristics of each isolated component, we view the physiological system as integrated oscillations in different time scales in which one modulation may be tightly affected by the others. In neuroscience, the interactions between activities in different time scales have been widely investigated to serve as a potential physiological biomarker. The origins of neuronal activities (i.e., discussions related to different cell population sizes and spatial scales) and their interactions (i.e., cross-frequency coupling and several hypotheses) are reviewed by Canolty *et al*. in detail^6^. In this paper, we focus on the existence and variation of *δ*-*α*/low*β* PACs due to the critical roles of these frequencies in consolidating memory and communication during sleep.

PAC is associated with neural coding as well as communicating within the local microscale and across the macroscale neural ensembles^6–9^. High-frequency oscillations, or the spiking rates of individual neurons^6^ may modulate by the low-frequency rhythmicity, which are assumed to regulate information between spatial regions by modulating excitability of local ensembles^10^. PAC thus facilitates effective coupling among neurons with similar shared phase, and high-frequency oscillations under specific phases of the slower waves are strengthened and synchronized^11^. PAC between high- and low-frequency activities was found stronger in the seizure onset zone than normal regions^12^. Nevertheless, the role of PACs under vigilance states during sleep has seldom been reported. Pathophysiological interferences in electrocorticography (ECoG) and local field potential (LFP) may be the reason for the limited reports. We aim to develop a reliable method in quantifying PACs of phasic events through the non-invasive measures EEGs: (1) a novel method masking phase-amplitude coupling (MPAC) is proposed to handle with the disorganized neurons and artifacts which may erase the observation of significant PACs in EEGs. (2) Only the large spatial scale of synchronization is considered in which these PACs may still reliable despite of the attenuation and desynchronization in layers. The strength of coupling may alter under different sleep states of vigilance and/or pathophysiological conditions, thus it should be carefully examined to avoid misinterpretation of the physiological dynamics. In an attempt to understand whether coupling is different in more or less synchronized phase-A subtypes, we focus purely on the physiological interaction between high-frequency (*α* and low*β* activities: 10–17 Hz) and low-frequency (*δ* activities: 0.25–2.5 Hz) bands with the consideration of interferences from sleep stages and pathophysiological factors. MPACs among different sleep stages and pathophysiological conditions are carefully examined and compared under various phase-A subtypes as well.

## Results

### Time-frequency analysis and phase-amplitude frequency plane

**Figure 1:**
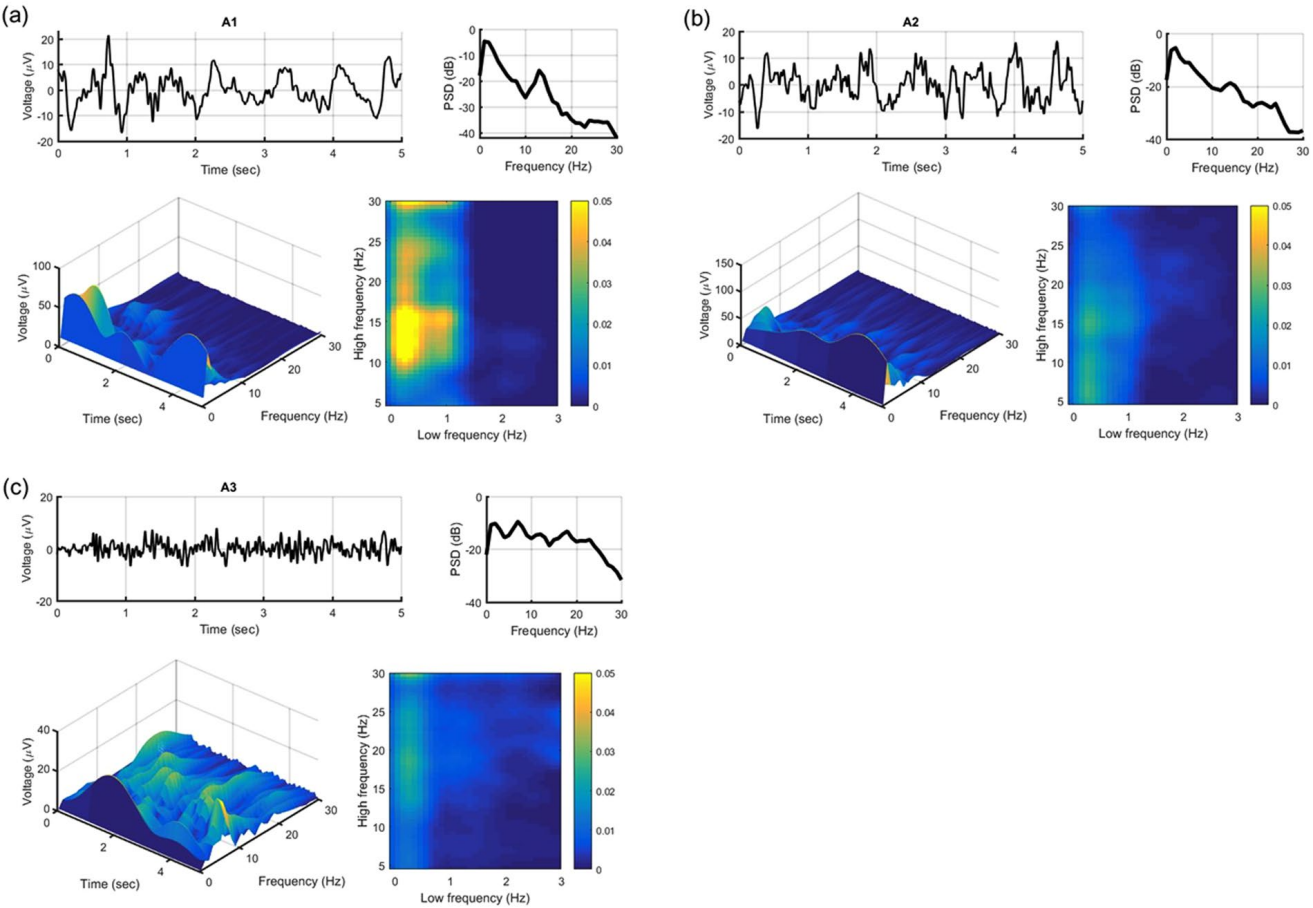
Spectral analysis, scalogram and comodulogram are demonstrated in different phase-A subtypes, including (**a**) A1, (**b**) A2 and (**c**) A3. A1 shows weaker high*β* frequency and stronger *α*/low*β* PAC.

In Fig. 1, power spectral density (PSD) is computed up to 30 Hz to see different spectra among phase-A subtypes. To estimate PSD, we use the Welch method in 1-sec Hann windows with 50% overlap. Frequencies below 30 Hz are normalized by summation of frequencies in the same range. Continuous Wavelet transform (CWT) is also applied and presented as 3D scalogram for better visualization in time-frequency resolutions. We aim to examine whether phase-amplitude frequency plane could explain any variance in phase-A subtypes independently from spectral power alone. In PSD and scalogram, high*β* power is the lowest in subtype A1 (−41 dB ~ −34 dB) (Fig. 1a) and the highest in subtype A3 (−32 dB ~ −16 dB) (Fig. 1c). In phase-amplitude frequency plane, the majority of subjects show stronger *δ*-*α*/low*β* PACs in subtype A1 (*MI* = 0.05) (Fig. 1a) than the other two (*MI* = 0.03). Interestingly, *δ* phase modulates mainly *α*/low*β* amplitude in subtype A2 (<18 Hz) (Fig. 1b), although relatively weak compared to A1, performs differently from subtype A3 (>15 Hz) (Fig. 1c) in which its amplitude is more prominent in *β* band.

### Phase-amplitude dependence

**Figure 2:**
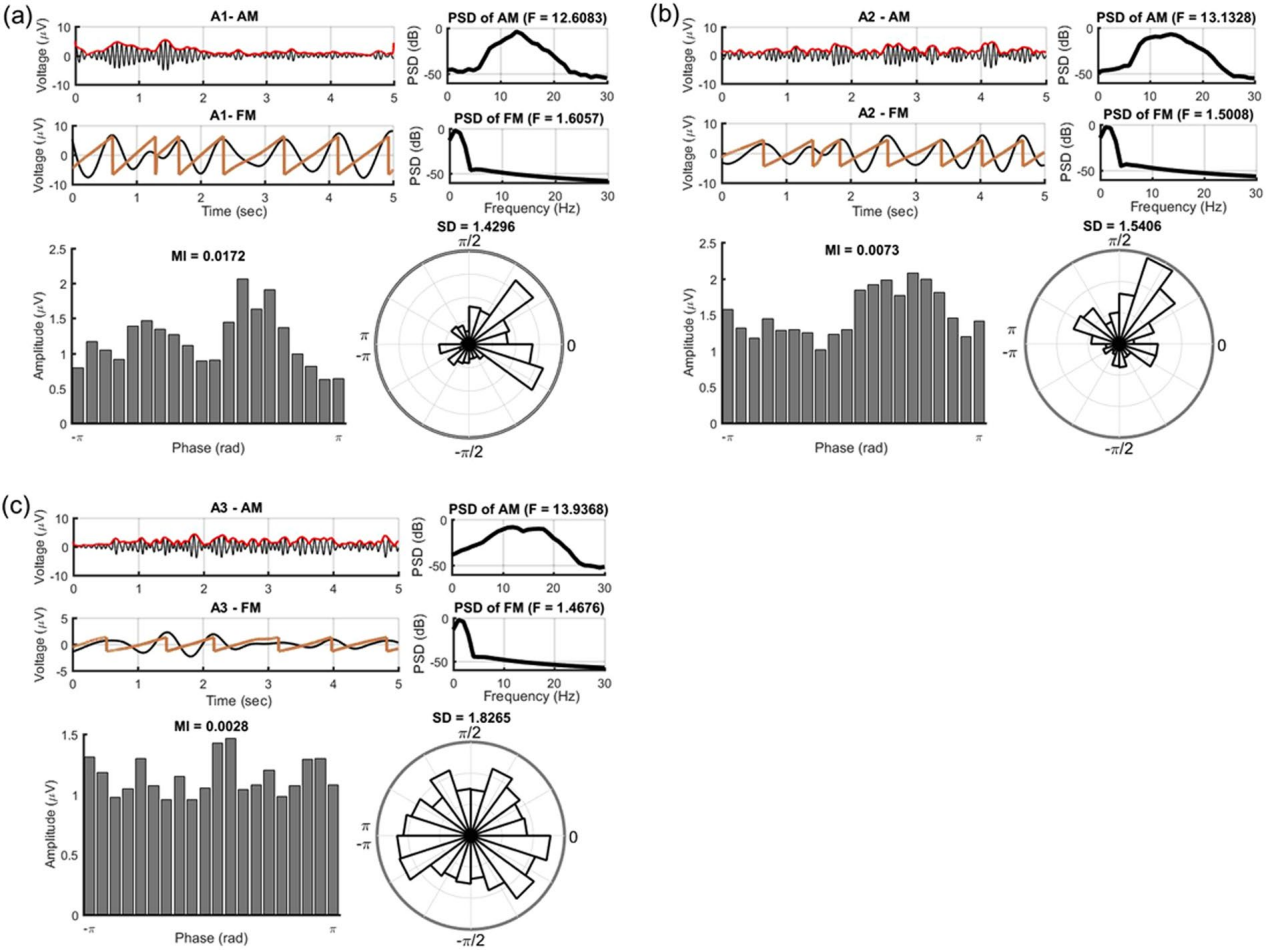
The phase-amplitude histogram shows *α*/low*β* amplitude distributed over *δ* phase in subtype **(a)** A1, **(b)** A2 and **(c)** A3. Both *δ* phase and *α*/low*β* amplitude which constitute the phase-amplitude distribution are provided along with the corresponding PSD. Subtype A1 shows higher normalized *α*/low*β* amplitude and larger MI (*MI* = 0.0172). Histogram chart in polar coordinate shows the distribution of phase difference between *δ* phase and *α*/low*β* amplitude. Subtype A3 show less phase-lock and larger SD (*SD* = 1.8265) than the other two.

Based on the images of phase-amplitude frequency plane, we concentrate on the association between the *δ* phase and *α*/low*β* amplitude in the following analyses. Whether *δ* phase and *α*/low*β* amplitude associate differently or not under various phase-A subtypes is critical. In Fig. 2, examples of *δ* and *α*/low*β* components in different phase-A subtypes are shown with the corresponding PSDs. As for the phase-amplitude distribution, *δ* phase modulates the most prominent *α*/low*β* amplitude in subtype A1 (*MI* = 0.0172) (Fig. 2a). Subtype A3, in contrast, shows relatively uniform distribution and the smallest MI (*MI* = 0.0028) (Fig. 2c). Histogram chart in polar coordinate which shows the distribution of phase difference between *δ* phase and *α*/low*β* amplitude is introduced to examine the alignment between these two frequencies. Both subtype A1 (Fig. 2a) and A2 (Fig. 2b) show relatively consistent phase-shifts between *δ* phase and *α*/low*β* amplitude compared to subtype A3 (Fig. 2c). Resulting in the larger standard deviation (SD) of phase difference between *δ* phase and *α*/low*β* amplitude in subtype A3 (*SD* = 1.8265) than the other two (A1: *SD* = 1.4296; A2: *SD* = 1.5406).

### Subtype A1 shows higher *δ*-*α*/low*β* MPACs

**Figure 3:**
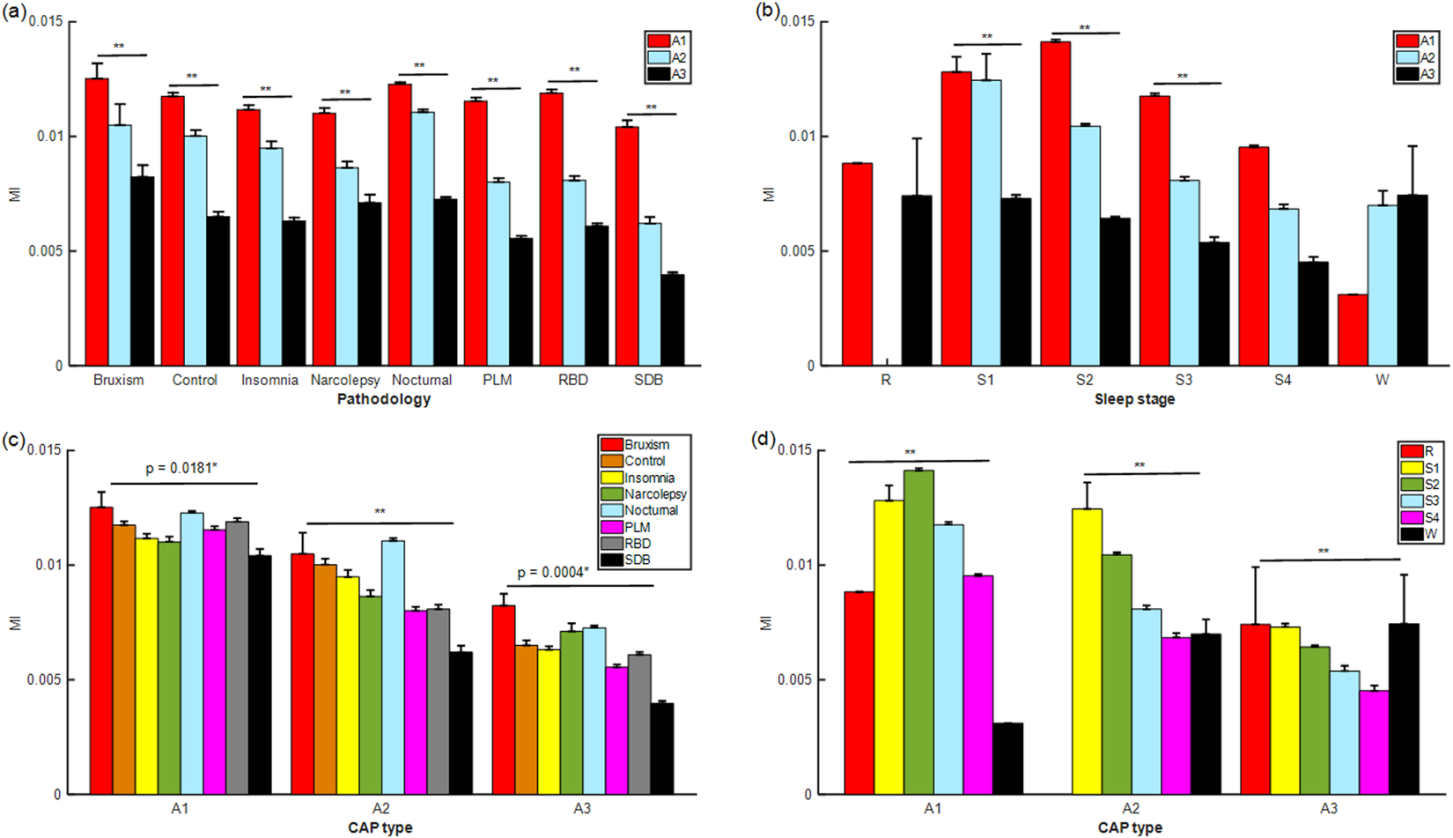
Statistics of *δ*-*α*/low*β* MPACs among phase-A subtypes are examined and grouped by (**a**) pathological conditions and **(b)** sleep stages. All pathological conditions and sleep stages show significant differences (*p* < 0.0001) among phase-A subtypes except S4. REM and Wake stages are not considered because of the insufficient samples. Significance test on *δ*-*α*/low*β* MPACs among **(c)** pathological conditions and **(d)** sleep stages grouped by phase-A subtypes are tested. Significant differences exist among pathological conditions and/or sleep stages in all three phase-A subtypes (** represents *p* < 0.0001). The error bars represent the standard error of mean (SEM). The abbreviation of pathologies and number of participants are summarized in Supplementary Table S1. The n value for each statistical analysis are provided in Supplementary Table S2.

Generalized linear mixed model (GLMM) is conducted to examine the effect of phase-A subtypes (A1, A2 and A3) on *δ*-*α*/low*β* MPACs under various pathophysiological conditions or sleep stages. We expect an amount of variability in the precise location of the contacts, subjects, pathophysiological conditions as well as the depth of sleep. Nevertheless, we decide not to bias results by pre-selection. We thus set these factors, if not grouped, as random factors in GLMM. Significant differences among phase-A subtypes appear in all pathophysiological conditions (*p* < 0.0001) (Fig. 3a). In addition, sleep stages include S1, S2 and S3 show the significant results (*p* < 0.0001) as well (Fig. 3b). No significant result is found in S4. REM and Wake stages are not considered because of the insufficient samples. Then we use the post hoc tests (*α* = 0.05, Tukey’s HSD) for pairwise comparison, indicating that *δ*-*α*/low*β* MPACs in subtype A1 is stronger than A2, and A3 is the smallest. This is a prevailing fact across all the aforementioned groups with significant results in GLMM.

### Effects of pathophysiological factors on *δ*-*α*/low*β* MPACs

*δ*-*α*/low*β* MPACs among pathophysiological factors grouped by phase-A subtypes are provided in Fig. 3c. There are significant differences among pathophysiological conditions in all three phase-A subtypes (A1: *p* = 0.0181; A2: *p* < 0.0001; A3: *p* = 0.0004). Combining the results of multiple comparison tests (*α* = 0.05, Tukey’s HSD) in all three phase-A subtypes, the effects of pathophysiological factors on *δ*-*α*/low*β* MPACs are validated. We summarize the effects of pathophysiological factors, which are generally consistent in all phase-A subtypes, from the largest *δ*-*α*/low*β* MPACs to the smallest: (1) Bruxism and nocturnal frontal lobe epilepsy. (2) Control and Narcolepsy. (3) Periodic leg movements, REM behavior disorder and Insomnia. (4) Periodic leg movements and Sleep-disordered breathing.

### Light sleep shows stronger *δ*-*α*/low*β* MPACs than deep sleep

Similarly, *δ*-*α*/low*β* MPACs among sleep stages grouped by phase-A subtypes are provided in Fig. 3d. There are strong significant differences among sleep stages in all three phase-A subtypes (*p* < 0.0001). In post-hoc test (*α* = 0.05, Tukey’s HSD), light sleep (mainly in S2) shows stronger *δ*-*α*/low*β* MPACs than the deep sleep (A1: S2 and S1 > S3 > S4; A2: S1 > S2 > S3 > S4; A3: S1 > S2 > S3). REM and Wake stages are widespread across all groups.

## Discussion

Many studies of brain activities indicate that PACs might play an essential role in neuronal computation, learning and communication^6^. In particular, evidences hint that pronounced changes in PACs may exist during sleep since sleep macrostructure was shown to be associated with the mechanisms of different frequency modulations^13^. Originally, the performances of seizure-prediction methods were shown to be affected by the EEGs in frequency domain^14^. In the recent years, significant changes in coupling under different sleep stages have been found, even in brain regions that are far away from the seizure zone^12^, which diminish the contributions from seizure foci^15,16^. This implies that there may exist some physiological PACs that are isolated from the pathophysiological matters. Due to the growing interests in sleep microstructure, we investigate the normal and abnormal sleep physiologies and compare PACs under phase-A subtypes in CAP. Normally, subjects that using intracranial electrodes (LFP or ECoG) are suffered from certain brain disorder, such as epilepsy or Parkinson’s disease, and should thus be treated carefully to avoid misinterpretation in physiological PACs. To this end, we develop an algorithm to quantify PACs using scalp EEGs due to its prevailing use in clinic, and CAPs may be an appropriate candidate to uncover the multiscale interactions in scalp EEGs for their high vigilance in amplitudes and modulations among phase-A subtypes.

Although evidences show that cross-frequency coupling (CFC), PAC in particular, is an elegant solution for various of neurological explanations, spurious CFCs may lead to misinterpretations in the true modulations between oscillations in different time scales due to the irregularity of physiological oscillations^17^. A comprehensive review on the concerns of the methodologies to cope with CFCs were summarized by Aru *et al*. in 2015^18^. EMD, as a method to extract the components with physical meaning on the time domain in non-static process, can reflect morphological changes effectively, and thus is applied to provide promising oscillations for PAC’s computation^19^. To address the issue of mode mixing when performing EMD, the phase-steps ranged masking technique of an equivalent dyadic filter bank structure is introduced. Having such a framework bring several benefits: (1) Applicable to complex system: some previous proposed masking methods add a pure-tone sine/cosine signal^20^ or further applicable to the synthetic signal comprising multiple pure tones with the help of the observation in Fourier spectrum^21^. By extracting dyadic patterns, no prior knowledge of the analyzed signals is required. (2) Low computational complexity: the masking method avoids the repeated computation like ensemble-EMD (EEMD) in reducing the effects of noise-aids^22^. (3) Reduce the incomplete decomposition: the phase-steps ranged approach can suppress the effect of additive sinusoidal wave more than using the two oscillations in opposite direction^20^. To quantify PACs between decompositions, Tort *et al*. compared potential indexes using simulated signals under different levels of challenges, and concluded that the modulation index (MI) showed the most satisfaction to cope with PACs^23^. We follow this usage in the study. The influence of signal-to-noise ratio (SNR), which may cause a deviated estimation over the accuracy of phases as well as PACs, is regarded as a complicated confounding factor in PAC computation. However, the distinct patterns of significant PACs can be destroyed by permuting the order of trials, as often seen in non-parametric tests, for which phase and amplitude are extracted^24^. The resulting blocks are determined according to their cycle frequencies in this study. Therefore, the time dependence between two series is disrupted but the nature of the physiological signal, i.e., the intra-wave modulation, is remained^25^. Thereby, the significant PAC measure can be observed if existed using surrogate data testing (see Method section for details). In addition, the reassignment of the frequency-frequency scatter plot can be achieved with cycle frequencies as well.

PACs in polysomnographic (PSG) are dominated by large-scale oscillations. Thus, we only focus on the frequencies below 30 Hz. Note that some amplitude frequencies, such as *γ* range or HFO, need not to be considered since they mainly appear in the LFP or ECoG records. Our findings indicate that *δ* phase modulates *α*/low*β* activity during sleep dynamically. Our findings suggest that PACs may serve both as a physiological or pathophysiological mechanism. In Fig. 1, the strongest high*β* power is found in subtype A3 (−32 dB ~ −16 dB) while subtype A1 (−41 dB ~ −34 dB) shows the weakest high*β* power but possesses stronger *δ*-*α*/low*β* PAC (*MI* = 0.05) than the other two phase-A subtypes (*MI* = 0.03). One intriguing explanation for why exaggerated levels of *δ*-*α*/low*β* PACs in sleep may easier to be observed in subtype A1 is that the *α*/low*β* oscillation become locked by the synchronization in high-voltage *δ* activities, and thus the initiating firing patterns underlying high*β* frequency and/or higher are prevented. In contrast, the desynchronization in high*β* activities in subtype A3 were more likely to disrupt the phase-amplitude dependence between *δ* and *α*/low*β* wave. Hence the reduction of coupling could free up resource for *α*/low*β* activity. In the light of the concept that the more constrained neurons in their firing patterns which bind within *α*/low*β* band could cause a high level of *α*/low*β* power, we could thus speculate that the amplitude and/or power of *α*/low*β* oscillations can bring about the *δ*-*α*/low*β* PACs. However, individual power do not overlap exactly with phase-amplitude frequency plane in *α*/low*β* peak frequencies, indicating that it is the interaction of *δ* phase with the amplitude of *α* or low*β* oscillations that lead to higher PAC estimate, but not the level of *α*/low*β* power. These differences among phase-A subtypes valid across groups in pathophysiological conditions (*p* < 0.0001) (Fig. 3a) and NREM sleep stages (p < 0.0001 except S4) (Fig. 3b). When comparing *δ*-*α*/low*β* MPACs among NREM sleep stages (Fig. 3d), light sleep shows stronger *δ*-*α*/low*β* MPAC than deep sleep (*p* < 0.0001) in all three phase-A subtypes. Note that S2 is more representative than S1 in light sleep since most of phase-A sequences are categorized in S2 instead of S1 (Supplementary Table S2). Besides, similar order in *δ*-*α*/low*β* MPACs among pathophysiological conditions (Fig. 3c) are shown across phase-A subtypes which manifests that both physiological and pathophysiological factors can contribute to *δ*-*α*/low*β* MPACs with certain independency.

Previous studies suggested that *β* power in PSGs may decrease as the depth of sleep increasing, and *δ*, *θ* and *α* power may increase in contrast. In our study, we extend these finding with the observation of *β* oscillations is largely confined to the high*β* range. This further strengthens the possibility that there might be a functional subdivision within the *β* band. *β* activity at lower frequencies may be more conspicuously related to the local computation in the cortex; whereas communication between LFP and ECoG seems to be mediated by high*β* frequencies in contrast^26^, our results suggest that the increase in potential information coding is the consequence of high*β* desynchronization which is coincide with the information theory resurgence recently^27^. For example, *δ* phase will phase-lock to the *α*/low*β* amplitude while the high*β* oscillations are suppressed and desynchronized (e.g. subtype A1).

The phase differences between coupled oscillations is critical in affecting local computation. For example, LFPs were observed to process faster or more comprehensive in stimuli that time-locked to the trough as compared to the peak, which suggests that high neuronal excitability is associated with the trough of an LFP oscillation^10^. In this paper, Fig. 2 shows stronger phase-dependent relationship between *δ* phase and *α*/low*β* amplitude in subtype A1, and thus implies the stronger communicated ability and wide-range synchronization. To reveal the functional role of *δ* activity, surgical outcome in epilepsy patients was found related to the slowness of inter-ictal *δ* activity. For some patients with epilepsy, correlation in slow wave background EEG activity were observed on seizure onset zone^28^. PACs have often been associated with information processing across the brain regions^7^. All of these suggest that *δ* activity can play a vital role in communicating along with the activities/information in its relatively higher frequencies carried.

In the present study, we propose a novel method in quantifying PACs between *δ* phase and *α*/low*β* amplitude using masking signals. More information (e.g. *δ*-*α*/low*β* PACs and their phase differences) can be obtained in addition to the traditional time-frequency analysis (e.g. PSD or even the nonstationary analysis like Wavelet transform). We suggest the MPAC can be a promising tool to quantitatively estimate the intensity of PACs.

## Method

### Materials

The all-night PSG recordings are downloaded from “The CAP Sleep Database” of “PhysioBank” (https://physionet.org/physiobank/database/capslpdb/)^2,29^. A collection of 108 PSG recordings with seven sleep-related pathologies plus normal healthy were included. Informed consent was obtained from all participants. 16 healthy subjects included in the analysis did not present any neurological, psychiatric and medical disorders. To avoid the affection of the central nervous system as much as possible, no drugs intake and excessive alcohol or coffee consumption were allowed for all these participants preceding the PSG recording. All experimental protocols were approved and registered at the Sleep Disorders Center of the Ospedale Maggiore of Parma, Italy^2,29^. The number of participants in each pathology are provided in Supplementary Table S1. The clinical information of all these 108 subjects can refer to the excel file (gender-age.xlsx) in the aforementioned link of this database. All methods were carried out in accordance with relevant guidelines and regulations^2,30^. Well-trained neurologists scored the sleep macrostructure based on the Rechtschaffen & Kales rules (sleep stages 1–4, wake, REM sleep and movement artifacts)^30^, whereas the CAP was determined according to the Terzano’s reference atlas of rules (phase-A subtypes include A1, A2 and A3)^2^. Segments annotated with movement artifacts (MT) are excluded in this study. The EEG traces were sampled at 100 Hz or 512 Hz. All materials, data sets and methods are available upon request.

### Signal preprocessing

To eliminate the effects from high-frequency noise and low-frequency movement artifacts in EEGs, a 6^th^-order Butterworth bandpass filter with cutoff frequencies set at 1 Hz and 50 Hz is adopted at first. Then, Principal Component Analysis (PCA) is used for dimensionality reduction without losing critical information. Principle components (PCs) in descending order with at least 95% of accumulated percentage of eigenvalues or variance are kept^31^. To reject EEG contaminations from eye movements, eye blinks and cardiac rhythms, Fast Independent Component Analysis (Fast ICA) is applied to estimate the independent components (ICs) of EEGs using the reconstructed EEGs after PCA^32^. ICs with correlation to EOG and/or ECG larger than 0.5 are rejected. By using PCA prior to ICA, the dimension reduction prevents overlearning which may sometime occur in ICA^33^, as well as the interrater error due to visual inspection.

### Masking decomposition

**Figure 4:**
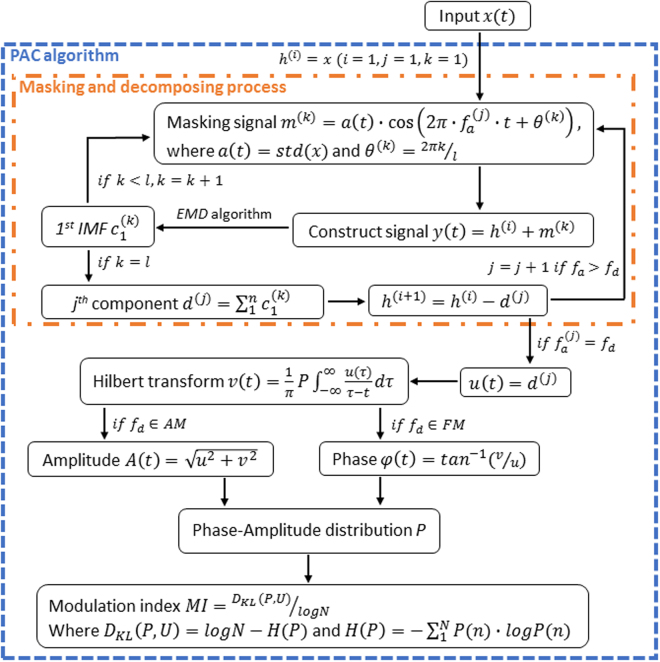
MPAC algorithm.

Empirical Mode Decomposition (EMD) is known for its ability of faithfully presenting irregular composition^19^. A simulated demonstration and the detailed algorithm of EMD is provided in the Supplementary Information. In this study, we generate the *δ* and *α*/low*β* activities from the total length of the analyzed sequences using the Fast EMD^34^ to reduce the amount of computation. However, EMD suffers from some weak points, and “mode mixing”, the oscillations of different frequencies coexist in one IMF due to intermittency^22^, could be the one that needs to be addressed the most. To make up for the deficiency, the masking technique was introduced to resolve mode mixing issue due to the intermittency^20^. In this study, the masking signals are designed with phase-steps ranged equally across a complete cycle. The technique aims to reduce the risk of incomplete cancellation due to deviated decomposition which may easier occurred in the previous masking technique with reverse wave superposition^20,21,35^. As indicated by the “Masking and decomposing procedure” in Fig. 4, masking signal with amplitude level equals to the standard deviation of the input is used to create a constructed signal, then the 1^st^ IMF is extracted using EMD algorithm. After recursively proceeding this process with different phase-stepped masking signals, the 1^st^ extracted component equals to the summation of all the 1^st^ IMFs. Similarly, the rest of the lower-frequency components are pursued using the dyadic-frequency masking signals.

### Modulation index

Among the work by Tort *et al*. which summarized and compared several methods of exploring PACs^23^, modulation index (MI) showed the best performance. To this end, we first obtain the analytic form of the interested signals with the help of Hilbert transform. The high-frequency amplitude and low-frequency phase is computed afterward. A phase-amplitude distribution is then constructed by first dividing the phase domain (0, 2*π*] into *N* bins (we use *N* = 20). High-frequency amplitude of each sample is assigned to a bin according to the low-frequency phase occurred at the same time. Averaging these amplitudes within each bin, and dividing by the total averaged amplitude yields the proportion of high-frequency amplitude occurring within each phase bin. The Shannon entropy *H* is computed by the equation $H(P)=-\sum _{j=1}^{N}P(j)\mathrm{log}\,[P(j)]$. Kullback-Leibler (KL) distance, defined as ${D}_{KL}(P,U)=\,\mathrm{log}\,(N)-H(P)$, is then divided by the maximum possible entropy log(*N*), yields a quantity between zero and one, defined as MI. “PAC algorithm” in Fig. 4 shows the flowchart of PAC quantification in this section.

### Cycle-Frequency

For each IMF, we calculate frequencies by cycles^25^. To estimate the cycle frequencies, we allow the phase series *φ*(*t*) to decrease only in increments smaller than *π*/4 to prioritize monotonicity. The transition between cycles are defined as the time points at which the unwound phase series cross integer increments by 2*π*. We calculate the cycle-frequency series *f*(*s*, *t*) that starts the frequency of the cycle from time point *s* and ends at time point *t*; i.e., $f(s,t)=(\frac{1}{t-s})(\frac{\phi (t)-\phi (s)}{2\pi })$ Hz for all the time points between *s* and *t*. The instantaneous phase series can be replaced by cycle-frequency series. Unlike the instantaneous frequency which may be affected by the waveform distortion, such as the abrupt high-frequencies caused by sharp peaks, the cycle-frequency is relatively robust to these factors. Meanwhile, the cycle-frequency can change adaptively, unlike a smoothed instantaneous frequency, it allows us to concentrate on the inter-wave phase modulation.

### Surrogate data testing

To test the significance of MIs if possesses unique temporal modulation, an approach for generating surrogate data is introduced based on the work by Pittman-Poletta *et al*.^25^. Briefly, the order of the resulting blocks is permuted independently for both the phase and amplitude series in which the locations of the cuts are determined based on their cycle-frequencies. Hence, the temporal relationship between high-frequency amplitude and low-frequency phase is disrupted with only the intra-wave modulation within each individual cycle remain intact. To both reduce the differences in MIs due to different phase-A durations as well as the impacts of artifacts in large time scales, only the sequences with more than two cycles in *δ* activities are included in the analyses. This process is repeated 100 times for each pair of IMFs in this study. By thresholding the *z*-scores of MIs using a normal distribution, significance level below *α* = 0.05 are rejected with Bonferroni-correction.

### Visualization of phase-amplitude frequency plane

Comodulogram is a direct visualization that allows the researchers to take a glance of the picture for which frequencies exhibit strong coupling. To guarantee high-frequency resolution, we first scatter the MIs associated with a pair of IMFs to multiple locations in the phase-amplitude frequency plane, based on their frequency coordinates. Each frequency coordinate is assigned a value equal to the significant MI. The redistributed MIs over the rectangular patches of phase-amplitude frequency plane are then averaged. In this paper, we averaged scatter plot within each 0.1-Hz phase frequencies and 0.5-Hz amplitude frequencies. Spanning from 0.1 Hz to 3 Hz for phase frequency, and 5 to 30 Hz for amplitude frequency.

### Statistical Analysis

The analysis aims to examine the ability of *δ*-*α*/low*β* MPACs in distinguishing the phase-A subtypes under different sleep stages and/or pathophysiological conditions. Shapiro-Wilk W test is used for assessing normality. General linear mixed model (GLMM) with the effects of sleep stages, pathophysiological conditions, subjects and location of electrodes are set as random factors to avoid their interferences. Two-tailed p value < 0.05 is considered to be statistically significant (*α* = 0.05 for all hypothesis testing). We use Tukey’s honest significance test (Tukey’s HSD) to test all possible pairwise differences of means as the correction for multiple comparisons. Likewise, similar statistical approaches are also applied to test the differences among sleep stages or pathophysiological conditions under different phase-A subtypes. The physiological data is analyzed in MATLAB (MathWork, Natick, MA). All statistical analyses are conducted using JMP, a Business Unit of SAS.

### Limitations of the Study

One important limitation of measured rhythms must be borne in mind is that the database is downloaded from the open database, and thus specific discussions, such as the types of sleep-disordered breathing and the impacts of obstructive sleep apnea, require additional experimental designs. In addition, to secure sufficient resolutions in PSD and permutations in the surrogate data testing, as well as to reduce the impact of differences in phase-A durations on MIs, we only include the data with more than two cycles in *δ* activities. For those with shorter sequences which could be contaminated by artifacts in large time scale is beyond our scope.

## Electronic supplementary material

Supplementary Information
