# Supplementary material for: Identifying Phase-Amplitude Coupling in Cyclic Alternating Pattern using Masking Signals
Source: Sci Rep. 2018 Feb 8;8:2649. doi: 10.1038/s41598-018-21013-9 (PMC5805690; doi:10.1038/s41598-018-21013-9)
Supplement: Supplementary file 1 — Supplementary Information [file 41598_2018_21013_MOESM1_ESM.pdf]

Supplementary Information

Title: Identifying Phase-Amplitude Coupling in Cyclic Alternating Pattern using Masking Signals

Authors: *Chien-Hung Yeh* and *Wenbin Shi*

Definition of CAP sequence and phase-A subtypes

CAP is initiated by sequence of an abrupt frequency and/or amplitude shift (phase-A) that prominent from the regular sleep pattern. In other words, phase-A is characterized by the transient EEG variations with the brain activation ascend to a higher level compared to its background (recurred intervals ≤ 1min). Phase-B, in contrast, corresponds to an intermittent lower level of activation, recovers background activity and separates the phase-As. A complete CAP cycle is initiated from an phase-A and a phase-B close behind. Both the two kinds of phases can last from 2 to 60 seconds. At least two consecutive CAP cycles are required to compose a CAP sequence. Non-CAP episode is thus defined as the remaining NREM sleep which is not occupied by CAP sequences.

The three phase-A subtypes of CAP are classified according to their spectral assessments. A1 is dominated by high-voltage δ waves (0.5-4Hz), and A2 appears when rapid activities occur for 20-50% of the total activation time, whereas A3 is characterized by rapid activities, especially in β activity (15-30Hz), which can occupy more than the half of the total phase-A duration[1]. Alterations of phase-A subtypes have been reported on several sleep disorders, such as sleep apnea[2], insomnia[3], narcolepsy[4] as well as nocturnal frontal lobe epilepsy[5].

Supplementary Table S1. Abbreviation of pathologies and the number of participants

Pathological conditions	Code	Participant #
Normal control	Control	16
Bruxism	Bruxism	2
Insomnia	Insomnia	9
Narcolepsy	Narcolepsy	5
Nocturnal frontal lobe epilepsy	Nocturnal	40
Periodic leg movements	PLM	10
REM behavior disorder	RBD	22
Sleep-disordered breathing	SDB	4

Supplementary Table S2. n value for statistical analysis

Phase-A subtypes	A1	A2	A3
**Pathological conditions**			
Control	2741	829	820
Bruxism	370	66	254
Insomnia	1389	602	1077
Narcolepsy	1068	416	658
Nocturnal	10205	4120	4045
PLM	2024	977	1514
RBD	4201	1649	2949
SDB	497	261	1003
**Sleep stages**			
S1	102	78	1976
S2	8916	6395	9154
S3	5456	1689	792
S4	8019	756	373

Supplementary Table S3. Nonstationary synthetic signal

Analytic form	Input = $X_p + X_a$
Lower frequency rhythm	$X_a = \text{Real}[1.5e^{j\theta X_a(t)}]$, f_p is the cycle frequency of X_p
Higher frequency rhythm	$X_a = \text{Real}[1.5e^{j\theta X_a(t)}]$, f_a is the cycle frequency of X_a
Period and frequency of X_p	17 samples $\leq T_p \leq$ 24 samples or 25Hz $\leq f_p \leq$ 35Hz
Period and frequency of X_a	9 samples $\leq T_a \leq$ 11 samples or 55Hz $\leq f_a \leq$ 65Hz
Details	Sampling Rate f_s = 600Hz, Duration $\cong$ 10sec

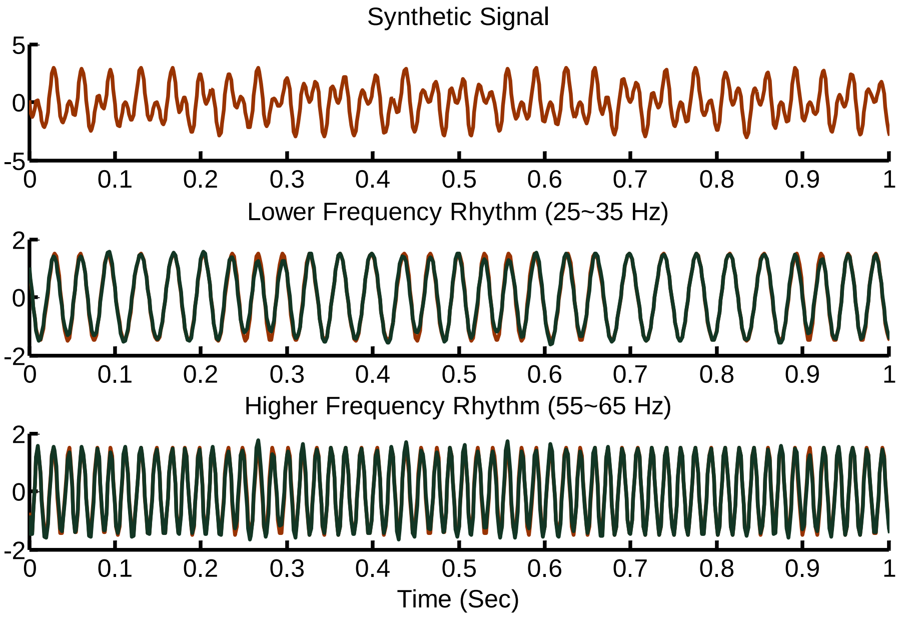

Supplementary Figure S1. Comparison between designed nonstationary components and IMFs.

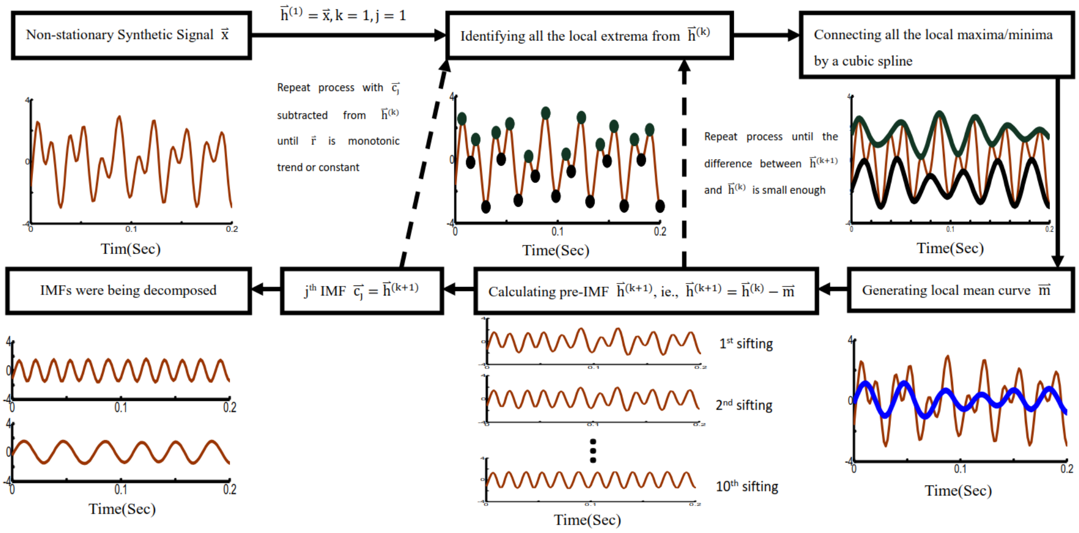

Supplementary Figure S2. EMD algorithm demonstrated using nonstationary signals

Empirical Mode Decomposition

Empirical Mode Decomposition (EMD) is a pre-processing method of Hilbert-Huang transform (HHT)[6]. EMD can decompose an inter- and intra-wave modulated time series into its intrinsic mode functions (IMFs), which are designed to obtain interested components based on the instantaneous spectra of IMFs. Due to its nature in maintaining the original shape of the signal, it is thus an adaptive and reliable tool in analyzing the physiological signals that may be nonlinear and nonstationary.

We design a synthetic signal to demonstrate the ability of EMD in handling with nonstationary composition. Two nonstationary components with frequencies range within 25~35Hz and 55~65Hz are designed (Supplementary Table S3). As shown in Supplementary Fig. S1, the extracted IMFs almost reproduce the designed components. Supplementary Fig. S2 shows the algorithm of EMD in steps. For a given signal $\vec{x}$, (1) Generating local mean curve: the algorithm begins with identifying all the local maxima and minima. The upper envelope $\vec{e_u}$ is generated by connecting all the local maxima using a cubic spline curve. Likewise, all the local minima are connected to create the lower envelope $\vec{e_l}$. Then we compute the mean $\vec{m}$ of these two envelopes. (2) Sifting process: the first component is obtained by subtracting $\overrightarrow{m^{(1)}}$ from $\vec{x}$, a qualified IMF should free of riding wave with its local mean curve close to zero at any point. The sifting process should perform again on $\overrightarrow{h^{(1)}}$ since $\overrightarrow{h^{(1)}}$ still possess multiple extrema between zero crossings. After recursively applying this step on $\overrightarrow{h^{(l)}}$, the sifting process will stop on the condition that the shortest period component of the signal (here we take the first IMF $\vec{c_1}$ as an example) is obtained. Then we separate $c_1(t)$ from the data and obtain the residue $r_1(t)$. (3) Generating IMFs: If the residue $r_1(t)$ still contains larger scales information, it is treated as a new input and repeated the sifting process again. This process should be repeated on all the subsequent residues. Finally, the EMD produce IMFs with a residue signal. Not until the residue $r(t)$ should either constant, or a monotonic slope, or a function with only one extremum that the whole procedure is terminated.

Reference

1. Terzano, M. G. & Parrino, L. Origin and Significance of the Cyclic Alternating Pattern (CAP). *Sleep Med.* **4,** 101-123 (2000).
2. Terzano, M. G. et al. Polysomnographic analysis of arousal responses in obstructive sleep apnea syndrome by means of the cyclic alternating pattern. *Clin. Neurophysiol.* **13,** 145-155 (1996).
3. Terzano, M. G. et al. CAP variables and arousals as sleep electroencephalogram markers for primary insomnia. *Clin. Neurophysiol.* **114,** 1715-1723 (2003).
4. Terzano, M. G. et al. Cyclic alternating pattern (CAP) alterations in narcolepsy. *Sleep Med.* **7,** 619-626 (2006).
5. Zucconi, M. & Ferini-Strambi L. NREM parasomnias: arousal disorders and differentiation from nocturnal frontal lobe epilepsy. *Clin. Neurophysiol.* **111,** S129-S135 (2000).
6. Huang, N. E. et al. The empirical mode decomposition and the Hilbert spectrum for nonlinear and non-stationary time series analysis. *P. Roy. Soc. A-Math. Phy.* **454,** 903-995 (1998).
